# The Impact of Close Reading Strategies on Individual Innovativeness and Life Skills: Preservice Teachers

**DOI:** 10.3390/bs14090816

**Published:** 2024-09-14

**Authors:** Yasemin Baki

**Affiliations:** Department of Turkish and Social Sciences, Faculty of Education, Recep Tayyip Erdoğan University, 53100 Rize, Türkiye; ysmnbaki@gmail.com

**Keywords:** close reading strategies, life skills, individual innovativeness

## Abstract

Reading is one of the fundamental tools for acquiring knowledge that has a direct effect on an individual’s life. Many single and multiple strategies are used to improve reading comprehension, which is a strategic acquisition. (1) This study aims to investigate the effects of close reading strategies on the life skills and individual innovativeness of preservice Turkish language teachers. (2) The study group for this research consists of 31 preservice teachers studying in the Turkish Language Teaching department at a university in northern Türkiye. A sequential mixed-method design was used. In the quantitative aspect of the research, a pre-test and post-test experimental design without a control group was used, while in the qualitative aspect, a case study design was employed. Data were collected using the Life Skills Scale, Individual Innovativeness Scale, and semi-structured interview form. In analyzing the data obtained to examine the effect of the experimental process, a *t*-test for dependent groups was used, whereas data from the interviews were analyzed through content analysis (3–4). According to the results obtained from the research, the close reading strategy was determined to be a strategy that significantly improves the life skills and individual innovativeness of preservice Turkish language teachers.

## 1. Introduction

Reading strategies, which are categorized as behaviors used by readers to direct their reading processes in acquiring, organizing, and structuring information, are tools used to discover the meaning of a text, facilitate understanding, and create a high level of understanding from a text [1]. Good readers use certain strategic behaviors and various strategies to discover the meaning of a text and facilitate this process [2,3]. Readers who act strategically in the reading process know how to read and cope with difficulties, regardless of the level of difficulty of the reading material they encounter, and how to monitor and regulate these strategies using metacognitive strategies, thereby facilitating the revelation of meaning in a text.

To facilitate reading comprehension and the development of these skills, readers must possess a certain proficiency, be familiar with reading comprehension strategies, and apply these strategies accordingly [4,5]. Although different definitions and classifications are made regarding strategies that facilitate reading comprehension, the most common classification is based on the stages of the reading process: pre-reading, while reading, and post-reading [6]. As readers encounter more complex texts thanks to the rapid changes in information technology and communication, multiple reading strategies (SQ3R, KWLS, PQ4R), formed by the joint and intertwined use of these strategies, are one of the important tools used in the reading comprehension process [1]. These strategies contribute to the development of reading comprehension skills and levels by increasing reading success [6,7,8]. These strategies aim to help the reader move from simple understanding to interpretive and critical understanding (Robinson & Good, 1987, as cited in [9]), because only when the reader possesses the skills that form these levels can they form deeper meanings in relation to the text [1].

While an in-depth understanding of a text is one of the ultimate goals of the reading process, it does not receive much attention in the educational process, which derives from the fact that standardized reading comprehension strategies are used in the education system [10]. Moreover, according to Roberts and Roberts [11], reading comprehension strategies are taught consciously to very few people within the framework of strategy instruction in education. Those who are successful in this are either intuitively or accidentally aware of them, so many students need assistance with learning these strategies in their educational lives. Therefore, even at the university level, it should not be assumed that students acquired advanced reading skills from high school, and the teaching of reading strategies should continue [12]. Moreover, research also suggests that university students do not use effective reading comprehension strategies [13,14]. In other words, although we have reached the digital information age and individuals can access all kinds of information at will, reading comprehension continues to be an undeniable reading literacy problem of the 21st century [10]. The impact created by digital tools complexifies texts, therefore requiring individuals to learn more strategies for reading comprehension than ever before. For this reason, teachers need to act as guides in the teaching and more effective use of reading strategies at every educational level [12], encourage reading, and learn and teach new strategies for the development of reading comprehension levels [11]. In addition, challenging texts are necessary for developing lifelong skills, and to analyze these texts, students need to work on reading difficulties with the support of a teacher, interact with books independently, and know the skills and strategies that will assist them in becoming lifelong readers [15,16]. It is necessary to benefit from new and original reading strategies in order to reveal meanings hidden in the inner layers of texts through polysemy and comprehend texts in depth.

The close reading strategy, one of the strategies used for deep understanding of a text, is a phased and multiple-part strategy that involves the intertwinement of many strategies that reveal and structure meaning in different layers of the text [17,18]. This strategy, which involves an analytical process that encourages deep thinking about the text [19,20], allows for the comprehensive examination of meaning in the text through multiple readings of a short, complex text, adding annotations to a text, discussing a text with questions based on a text, and thus examining the meaning in a text extensively through prior knowledge and personal experiences [21,22,23]. This repeated reading action with various strategies extracts the cognitive skeleton of a text, and group discussions reveal the meaning in a text [24,25]. These text-based questions and discussions allow for the resolution of connections between key words, the form of a text, the author’s style, rhetorical tools, imagery, word choice, and syntax in a text for close analysis [26,27], thereby facilitating a deeper understanding of a text [28]. This is because the close reading strategy develops strategic reading skills that contribute to critically examining a text [29,30,31].

Reading skills, which need to be continuously updated due to the value they add to life as a learning activity, are among the essential life skills an individual should possess in the 21st century [32,33]. According to Lapp et al. [34], deep reading strategies are believed to contribute to gaining knowledge about real life and making sense of relevant events. Deep reading is a necessary skill for success in all areas of life [12] since it encourages the reader to delve deeper into a text, enhancing inferential skills, decision-making abilities, and perspectives in today’s increasingly complex world [35]. This study also highlights the importance of integrating life skills into the curriculum and the necessity of assessing these skills. Various studies were conducted to ensure the integration of life skills into the curriculum [36,37,38,39,40]. Although various studies have been conducted to reveal the relationship between reading and life skills [32,41,42,43], no research has been found that examines the effect of the close reading strategy, which allows for a deep understanding of a text, on life skills. In reading studies, the primary goal is not just to measure comprehension skills but to understand how much of what is read is transferred to an individual’s life and what positive changes and innovations this transfer creates in their life.

Therefore, the education system should enable children to effectively cope with the environment, recognize existing opportunities, and deal with societal challenges. To activate these behaviors and overcome transformations in children, the school curriculum should be enriched with effective life skills, and learning outcomes on life should be observed [40]. This is because the new knowledge era of the 21st century, brought about by changing global conditions, has redefined priorities for schools, classifying skills into learning and innovation skills, life and career skills, and information, media, and technology skills [44]. Among these skills, innovation skills are also prominent. Innovation is the attitude, interest, desire, competence, and ability to implement innovation into life [45], with innovation defined as producing an original, new, and valuable product [46]. Innovation is a multidimensional concept consisting of various skills such as creativity, thought leadership, openness to experience, risk-taking, adaptation, acceptance, tolerance, and being open to new experiences [47]. Individual innovation can be defined as the attitude an individual develops towards creating, adopting, and implementing innovations and taking risks to realize these changes [48]. Individual innovation, which includes many concepts, classifies individuals or social structures into five categories, namely as innovators, pioneers, questioners, skeptics, and traditionalists according to the level and degree of the individual or social structure’s adoption of innovation [45]. These categories and their characteristics are as follows [49]:

1. **Innovators:** Innovators are individuals who are willing to try new ideas, curious, willing to take risks, enterprising and well educated, and have a vision.
2. **Pioneers:** People in this category, who come after innovators, support innovations and convey them, and are role models and guides in society regarding innovations.
3. **Questioners:** These people, who come after pioneers in supporting innovations, adopt innovations, but are cautious about implementing innovations and are not willing to take risks.
4. **Skeptics:** These people, who come after questioners, are more skeptical than average individuals in adopting innovations; they remain hesitant towards innovations due to factors such as social pressure and economic reasons.
5. **Traditionalists:** These people, who are the last to adopt innovations, are prejudiced against innovations and change, do not want to go beyond their traditions, and tend to ignore and delay innovations by acting according to the perspective of the previous generation.

Individual innovation creates a dynamic power in the emergence of performance in society with the synergy it creates among individuals [50]. Innovative educational institutions play a key role in the training of innovative individuals who enable this power to emerge. Innovation in education is the design and management of each stage of the education process according to the interests, needs, and abilities of the students in order to train creative and innovative individuals who do not passively receive information but actively discover and create it, and who can transfer the innovations of the modern age to their lives [51]. Innovation in education allows for the inclusion of practices that will shape the future in education processes by transcending traditional methods and ensuring adaptation to change and development, as well as the emergence of new ideas and changes. Therefore, it is essential for teachers to be open to learning and innovations [52], which is indispensable in order for students to be nurtured and supported in every aspect [53]. It is necessary for teachers to possess individual innovativeness traits to enrich the learning process [54] and transfer these skills to their students [53]. Educational institutions are among the main centers that influence and are affected by change, while also determining practices and policies for change [55]. Teachers, as the main actors of these institutions, need to be open to innovation, which plays a significant role in the development and improvement of the learning process [54] because the quality of education cannot surpass the quality of teachers [56]. Therefore, teachers need to be the leaders of innovation in society, serving as talent scouts, inventors, and managers, inspiring their students [57]. Being open to innovations and learning is indispensable for nurturing and supporting students in every aspect [53]. Despite these skills being necessary for individuals in social life, many of them are not included in the higher education curriculum [38]. To ensure that students graduate with the skills needed in both their social and professional lives in the 21st century, these skills need to be learned and actively used both in formal education and teacher training processes [32].

Despite the positive effects on and contributions of close reading strategies to the reading process, existing research focuses on preschool, primary school, and middle school levels [19,31,58,59,60]. In addition, the majority of studies focus on reading and reading comprehension skills based on the deep reading strategy [26,59,61,62,63,64,65]. However, it is crucial to recognize that young people need more information on strategy use and to observe the effects of young people’s use of this strategy [60,66]. Current research demonstrates that teachers prefer well-known strategies at a moderate level rather than specific strategies in their fields [67], and that they face challenges in conducting close reading in lessons [68]. After implementing these strategies, it has been observed that the strategies used by teachers change and develop [67]. Thus, this study aims to examine the impact of deep reading strategies on the development of individual innovativeness and life skills of preservice Turkish language teachers. By identifying the strengths and weaknesses of this strategy, it is intended to effectively incorporate it into the higher education reading process and describe its effects on transferring this change to life skills and individual innovativeness. Within this framework, this study seeks to answer the following questions:

1. Do close reading strategies create a significant difference between pre-test and post-test scores on individual innovativeness of preservice Turkish language teachers?
2. Do close reading strategies create a significant difference between pre-test and post-test scores on life skills of preservice Turkish language teachers?
3. What is the distribution of pre-test and post-test scores on individual innovativeness according to gender?
4. What are the views of Turkish teacher candidates on the impact of using the close reading strategy on individual innovativeness traits and life skills?

## 2. Materials and Methods

### 2.1. Research Model

This study uses a mixed-method approach, combining qualitative and quantitative approaches [69,70]. This approach allows for the collection and analysis of both types of data, aiming to gain a close understanding of the research process and data through multiple perspectives offered by qualitative and quantitative paradigms. The mixed-method design used in this study is an explanatory sequential design. In this design, quantitative data are collected first, followed by qualitative data, which are used to explain the quantitative findings in more detail [70]. In this study, an experimental intervention was first conducted, and related data were collected. Following this, semi-structured interviews were conducted with participants in the test group to collect qualitative data, which supported and explained the quantitative findings, allowing for a comprehensive evaluation of the intervention results.

In the quantitative part of the study, a pre-test–post-test quasi-experimental design without a control group was used to examine the impact of the deep reading strategy on individual innovativeness and life skills. This experiment investigates the effect of experimental intervention through pre-tests and post-tests applied to the same groups before and after the intervention [71,72]. In this study, the experimental intervention was the close reading strategy, and the individual innovativeness and life skills of preservice Turkish language teachers were intervened. The stages of the experimental intervention used in the study are shown in Table 1.

In the qualitative part of this study, a case study design, which allows for detailed and close data collection and a comprehensive description of the case, was used. This design provides a detailed reflection of the current case through examining a segment of it without generalization [73]. Furthermore, semi-structured interviews were conducted with participants in the test group to collect qualitative data on their views on the impact of the close reading strategy on individual innovativeness and life skills.

### 2.2. Study Group

The subjects in the quantitative part of this study were determined using a simple random sampling method. The participants selected through this method consisted of 31 preservice teachers studying in the Department of Turkish Language Teaching at a university in northern Türkiye. Characteristics of the study group are given in Table 2.

For the qualitative part of this study, the sample was determined using the purposeful sampling method of easily accessible case sampling. Through this sampling method, the 31 preservice teachers involved in the experimental process also formed the qualitative study group. Thus, the effects of the close reading strategy on life skills and individual innovativeness were examined in the same group both qualitatively and quantitatively.

### 2.3. Data Collection Tools

The research data were collected using the Individual Innovativeness Scale, the Life Skills Scale, and a semi-structured interview form.

#### 2.3.1. Individual Innovativeness Scale

This measurement tool, developed by H. Thomas Hurt, Katherine Joseph, and Chester D. Cook to determine the individual innovativeness of teacher candidates, was adapted in Turkish by Kilicer and Odabasi [74]. The scale consists of 20 items and has a four-factor structure: “resistance to change”, “leadership in ideas”, “openness to experience”, and “risk-taking”. The overall Cronbach’s Alpha reliability coefficient for the scale is 0.82, and for the sub-dimensions of resistance to change, leadership in ideas, openness to experience, and risk-taking, the coefficients are 0.86, 0.86, 0.72, and 0.78, respectively. Based on these findings, the scale can be considered reliable both as a whole and in terms of its sub-dimensions [74]. Participants can be categorized in terms of innovation with the scores calculated on the scale. Accordingly, if the participants’ scores are above 80 points, they are evaluated as “innovative”, if they are between 69 and 80 points, they are evaluated as “pioneer”, if they are between 57 and 68 points, they are evaluated as “questioner”, if they are between 46 and 56 points, they are evaluated as “skeptic”, and if they are below 46 points, they are evaluated as “traditional”.

#### 2.3.2. Life Skills Scale

This measurement tool, developed by Bolat and Balaban [75] to determine the life skills of preservice teachers, consists of 30 items and five sub-factors: coping with emotions and stress, empathy and self-awareness skills, decision-making and problem-solving skills, creative thinking and critical thinking skills, and communication and interpersonal relationship skills. The overall Cronbach’s Alpha reliability coefficient for the scale is 0.90, and for the sub-factors, the coefficients are 0.82, 0.77, 0.72, 0.73, and 0.66, respectively. Based on these findings, the scale can be considered reliable both as a whole and in terms of its sub-dimensions [75].

#### 2.3.3. Semi-Structured Interview Form

In the study, a semi-structured interview form was used to examine the views of Turkish language preservice teachers regarding the contributions of close reading strategies to their reading process, individual skills, and life skills. To prepare the interview form, a literature review was conducted, and questions were drafted by the researcher. The initial version of the form contained four questions, which were reviewed by two different field experts. Based on their feedback, necessary linguistic adjustments were made, and two questions were removed. The form was then piloted with four teacher candidates studying in the Turkish language teaching program. The pilot study confirmed that there were no unclear aspects or potential issues in the form. The final questions included in the form are as follows:

1. What are the contributions of the close reading strategy to your reading skills?
2. What are the contributions of the close reading strategy to your life skills and individual innovativeness?

### 2.4. Data Collection

**Figure 1:**
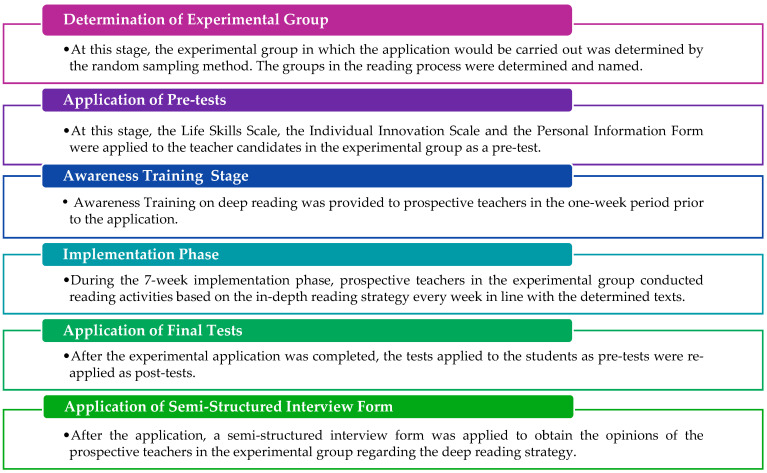
Experimental process.

The research involved collecting both qualitative and quantitative data in two phases. The entire experimental process lasted 11 weeks, including 7 weeks for the experimental intervention, 2 weeks for administering pre-tests and post-tests, 1 week for awareness training, and 1 week for semi-structured interviews which comprised the qualitative part. Pre-tests and post-tests were used to collect quantitative data for examining the impacts of the experimental intervention by means of the close reading strategy. These tests included the Individual Innovativeness Scale and the Life Skills Scale, which assessed the participants’ current conditions. For qualitative data collection, semi-structured interviews were conducted with participants in the test group. The steps of the experimental process are shown in Figure 1.

**Figure 2:**
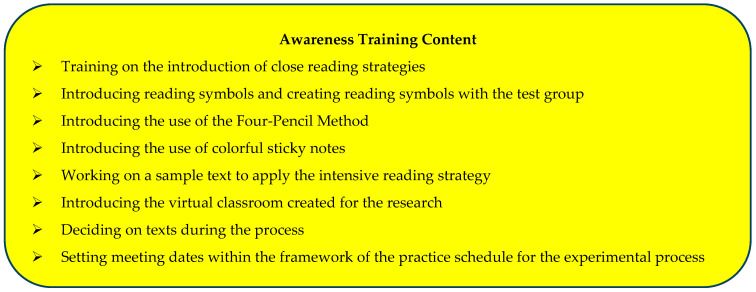
Awareness training.

Prior to practice, students in the test group were informed about the research. Participants were divided into groups and were asked to choose group names to facilitate the feasibility of the close reading strategy. The group names selected by the participants were Mars, Venus, Uranus, Jupiter, Pluto, and Mercury. Subsequently, pre-tests were administered, followed by awareness training in the close reading strategy. This training provided the necessary skills for using the strategy in the reading process and was delivered through practical practices. The content of this training is shown in Figure 2.

The following steps were taken during awareness training to teach the close reading strategy to preservice teachers in the test group (adapted from Beckman, 2002, as cited in [76]):

1. **Identification:** The instructor names the strategy.
2. **Teaching:** The purpose of the strategy is explained, including the benefits of learning the strategy and its importance.
3. **Usage Explanation:** Information on where the strategy can be used (e.g., while listening to a lecture, reading a text, post-reading) is provided.
4. **Usage Characteristics:** Explanation is provided of the characteristics of the strategy and what to pay attention to while using it.
5. **Modeling:** The teacher demonstrates the use of the strategy by thinking aloud, externalizing cognitive processes.
6. **Practice:** Students are given the opportunity to practice the strategy. During this trial phase, they can adjust their skills in a classroom setting prior to practice.
7. **Group Discussion and Evaluation:** Students evaluate and discuss their correct or incorrect use of the strategy as a group, encouraging them to monitor and assess their strategy use.

The awareness training lasted one week, comprising a total of six hours. To collect materials and ensure that participants could access necessary documents when needed, a virtual classroom was created where the steps of the strategy and practice examples were shared.

Not all texts are suitable for practicing the close reading strategy. It is recommended that the texts used for this strategy be checked for suitability considering student level and their compliance with the criteria for close reading [19,58,77]. Because of the complex nature of close reading, it is necessary to select the required texts based on participant preferences, participant level, length, complexity, difficulty level, etc. [78,79]. Any text type can be used for close reading. However, informational texts suit this strategy better as they typically contain more unknown words and concepts [77,78]. In addition to the needs and interests of the readers, the texts are also chosen according to the age-appropriate use of text types [10].

The texts chosen for this research were informational and philosophical texts, in line with participants’ requests, due to the focus on the impact of close reading strategies on life skills. Informational texts help students acquire direct knowledge about real life and understand social phenomena [31]. The book *Philosophy for Young People* [80] was selected considering the brevity and complexity criteria, and its content was deemed suitable (informational, including complex concepts, etc.) in consultation with two subject experts.

Of the 33 texts in the book, 10 were chosen based on participant interest through a survey. These texts were reviewed by the researcher for suitability using the Checklist for Selecting Texts for Close Reading [19,81] and the Readability Formula by Atesman [82] to determine difficulty level. All selected texts met the suitability criterion for close reading and were classified as “difficult”. Subsequently, six questions (three for deep understanding and three for simple understanding) were prepared for each text following established principles [77,83].

These texts and questions were reviewed for content and strategy suitability by two subject experts. After reaching a consensus, 7 out of the 10 texts were selected, and the questions were finalized with minor modifications. The texts used during the experimental process are presented in Table 3.

**Figure 3:**
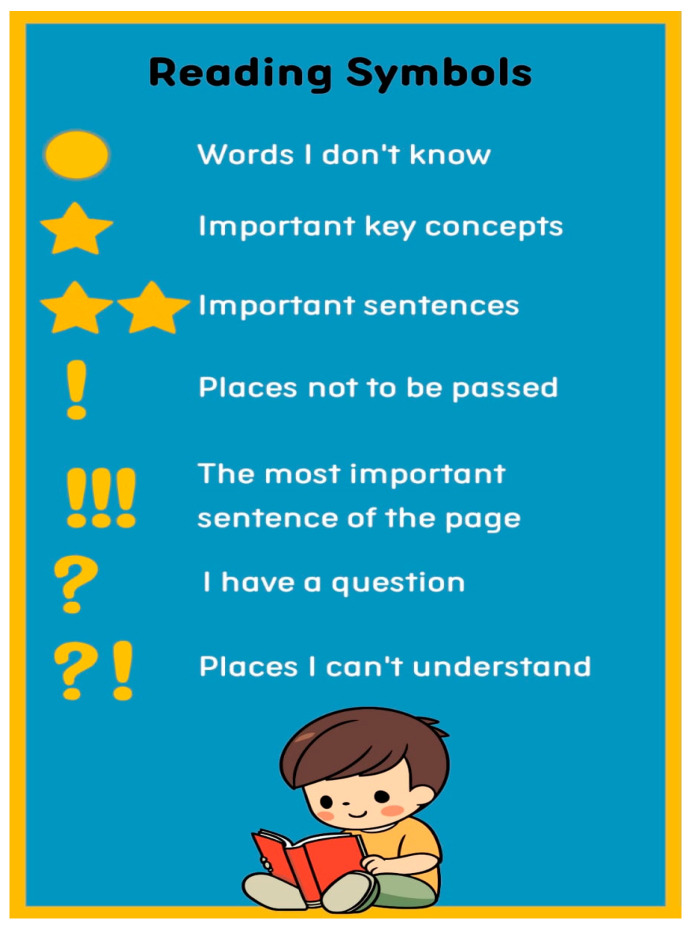
Reading symbols.

Following awareness training, weekly group meetings were held to read the selected texts while considering the close reading strategy. Each week, a different student was assigned as the reading leader to manage the reading process according to the prepared plan. The reading leader was responsible for managing the time for each phase and uploading student annotations onto the virtual classroom. Tools like “Reading Symbols”, the “4 Pen Method”, and colored sticky notes were used during this process. An example of the “Reading Symbols” card prepared with the students is shown in Figure 3.

**Figure 4:**
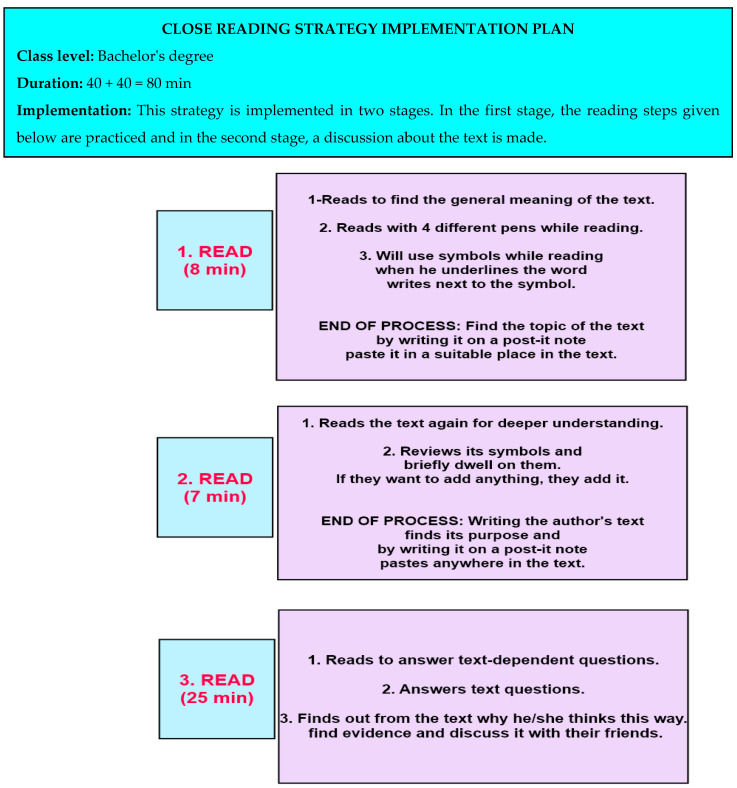
Example plan for close reading strategy [77].

Each strategy practice lasted two class hours, following these steps in Figure 4 [77]:

In the second class following the first, a discussion environment was created where students shared their thoughts. They discussed their annotations, the reasons for using specific symbols, and the ideas presented in the texts, providing evidence from the texts to encourage deep thinking. Post-tests were administered to the experimental group, and semi-structured interviews lasting approximately 30 min were conducted with all students in the experimental group. Sample images of the texts, which test group students read using the close reading strategy, are presented below.


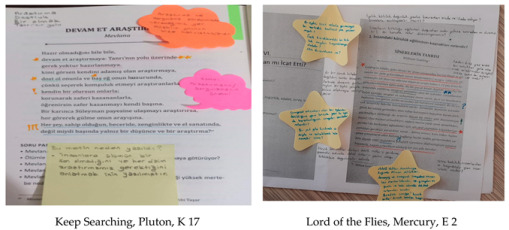


### 2.5. Data Analysis

This study employed a sequential analysis technique to analyze the obtained data [69]. Both quantitative and qualitative data from the study were analyzed sequentially, with dependent group *t*-test analysis used for the quantitative data from the Individual Innovativeness and Life Skills scales. Qualitative data obtained from semi-structured interviews were descriptively analyzed. In this analysis, the data from the interviews were subject to a close analysis, with the resulting codes categorized under certain themes [84]. Each question in the form was evaluated as a theme, and the data were analyzed based on these themes. To increase the reliability of the study, the consistency of the scores from analyses conducted dependently by the researcher and a field expert was evaluated as “agreement” or “disagreement”. In cases of discrepancies between the researcher and the field expert, a consensus was reached, and adjustments were made. The reliability of the data analysis coding was calculated using the formula [Agreement/(Agreement + Disagreement) × 100] [85]. This coding, conducted independently by the field expert at a different time, resulted in 92% agreement between the two coders, indicating high consistency and reliability.

## 3. Findings

This section presents the quantitative and qualitative findings related to the impact of the close reading strategy on the life skills of preservice Turkish language teachers under two separate headings.

### 3.1. Quantitative Findings on the Impact of the Close Reading Strategy on Life Skills and Individual Innovativeness Features

The *t*-test results comparing the pre-test and post-test scores of preservice Turkish language teachers on the Life Skills Scale are presented in Table 4.

According to Table 4, there was a significant difference in favor of the post-test scores ($\bar{X}$ = 114.83) compared to the pre-test scores ($\bar{X}$ = 105.03) in the total of the Life Skills Scale for the test group (t_(30)_ = 5.02, *p* < 0.05). Similarly, significant differences were found in favor of the post-test scores in the subscales of coping with emotions and stress (t_(30)_ = 2.99, *p* < 0.05), empathy and self-awareness (t_(30)_ = −2.81, *p* < 0.05), decision making and problem solving (t_(30)_ = −5.44, *p* < 0.05), creative and critical thinking (t_(30)_ = −2.90, *p* < 0.05), and communication and interpersonal skills (t_(30)_ = −4.27, *p* < 0.05). These findings suggest that the close reading strategy is a reading strategy that comprehensively enhances life skills in preservice teachers.

The *t*-test results comparing the pre-test and post-test scores of preservice Turkish language teachers on the Individual Innovativeness Scale are presented in Table 5.

According to Table 5, there was a significant difference in favor of the post-test scores ($\bar{X}$ = 70.67) compared to the pre-test scores ($\bar{X}$ = 64.51) in the total of the Individual Innovativeness Scale for the experimental group (t_(30)_ = −4.27, *p* < 0.05). Significant differences were also found in the subscale of resistance to change (t_(30)_ = 2.28, *p* < 0.05), but no significant differences were found in the subscales of thought leadership (t_(30)_ = −0.92, *p* > 0.05), openness to experience (t_(30)_ = −1.10, *p* > 0.05), and risk-taking (t_(30)_ = 0.43, *p* < 0.05). In the openness to experience subscale, no significant difference was found between the pre-test mean score ($\bar{X}$ = 18.29) and the post-test mean score ($\bar{X}$ = 19.35) in favor of the post-test scores (t_(30)_ = −1.10, *p* > 0.05). Similarly, in the risk-taking dimension, no significant difference was found between the pre-test mean score ($\bar{X}$ = 6.80) and the post-test mean score ($\bar{X}$ = 6.58) in favor of the post-test scores (t_(30)_ = 0.43, *p* < 0.05). These findings suggest that the close reading strategy significantly influences the overall individual innovativeness and resistance to change of preservice teachers but does not significantly influence thought leadership, openness to experience, or risk-taking.

The comparison of the descriptive statistics results for the pre-test and post-test scores of preservice Turkish language teachers by individual innovativeness categories according to gender is presented in Table 6.

According to the pre-test scores in Table 6, the majority of male teacher candidates were in the pioneer category, while the majority of female teacher candidates were in the inquisitive category. When evaluating the pre-test scores as a whole, it was observed that the majority of teacher candidates were in the pioneer and inquisitive categories. From the post-test results, it is seen that the majority of male teacher candidates were in the inquisitive and skeptical categories, whereas the majority of female teacher candidates remained in the inquisitive category. Considering the post-test scores as a whole, it can be said that the majority of teacher candidates were in the inquisitive category, followed by the skeptical category. Comparing the distribution of categories in pre-test and post-test scores, it was observed that no teacher candidate remained in the innovative or traditional categories following the experimental process.

### 3.2. Qualitative Findings on the Effects of the Close Reading Strategy on Turkish Teacher Candidates’ Reading Skills, Life Skills, and Individual Innovativeness

This section presents the qualitative findings on the contributions of the close reading strategy to the reading skills, life skills, and individual innovativeness of preservice Turkish teachers.

Table 7 shows preservice Turkish teachers’ views on the contributions of the close reading strategy to the development of their reading skills.

According to Table 7, when the contributions of the in-depth reading strategy to the reading skills of Turkish teacher candidates are examined, 100% stated that it improved their skills in analyzing the text in depth, 38.70% stated that it improved their understanding of the importance of using different strategies, 32.25% stated that it improved their skills in questioning the source and accuracy of the information in the text, 25.80% stated that it improved their reading comprehension skills, and 19.35% stated that it improved their questioning skills.

Participant views on the contribution to reading skill development were as follows:

*It helped me approach texts from different perspectives.*

(F, 1)

*It made me think critically and look at a text from different angles rather than taking it as it is.*

(M, 1)

*Reading using different techniques motivates a person. Now, when I read books, I will use techniques from different genres. This will give me different experiences.*

(F, 8)

*I realized that the texts I read have deeper meanings, not just one interpretation.*

(F, 17)

*The texts we read contributed to my change as they were disruptive to my stereotypical knowledge.*

(F, 19)

*Using the close reading strategy added a new style to my reading habits.*

(M, 3)

*Asking different questions and doing it step by step was fun and educational. Reading in detail to find answers to the questions in the text helped us understand the texts better.*

(F, 11)

*I can better find the cause-and-effect relationships and connections in the text.*

(M, 5)

Table 8 presents the views on the contributions of the close reading strategy to the life skills and individual innovativeness of Turkish teacher candidates.

According to Table 8, when the effects of the close reading strategy on the life skills of Turkish teacher candidates are examined, 100% stated that it improved their respect for differences, 96.77% their critical thinking and self-awareness, 83.87% their self-awareness, 67.74% their empathic thinking, 58.06% their decision making and evaluation, 61.29% their communication skills and interpersonal harmony, and 51.61% their multidimensional and deep thinking skills.

According to Table 8, when the effects of the close reading strategy on the development of individual innovativeness characteristics of Turkish teacher candidates were examined, 96.77% stated that it improved their skills in leading new ideas, and 41.93% their skills in being open to new ideas and experiences.

Participant views on the contributions to life skills and individual innovativeness were as follows:

*I learned to look at life from different angles, to derive meaning from everything that comes our way, and to realize that nothing happens by chance.*

(F, 5)

*I learned to see even a stone in my path as meaningful.*

(M, 3)

*It made me open to new ideas and listen to others’ thoughts with respect.*

(F, 18)

*I felt the need to question things after this practice.*

(F, 17)

*Now I look at things from a broader perspective and question them, and I am much more open to new ideas as an individual.*

(F, 21)

*Previously, I was even afraid to say the word ‘create’, let alone think critically about certain topics; I was afraid to think at all.*

(F, 4)

*I realized that I was thinking one-dimensionally and couldn’t look at events critically.*

(F, 6)

*I think it contributed to my development in problem-solving.*

(F, 11)

*Since it developed my questioning and thinking skills, my decision-making skills also improved along with it.*

(F, 8)

*With this strategy, I learned to better recognize my own thoughts and feelings.*

(F, 17)

*Now, I started to think more deeply about a topic, event, or text.*

(F, 25)

*I now communicate according to the characteristics of individuals.*

(F, 19)

*It was beneficial in terms of decision-making and problem-solving.*

(M, 1)

*I once again realized how necessary it is to see from others’ perspectives.*

(F, 15)

*It helped me recognize my own thoughts and feelings.*

(M, 2)

## 4. Conclusions and Discussion

According to the results obtained from the research, it can be said that the close reading strategy has a significant effect on the development of life skills of Turkish teacher candidates. According to Lapp et al. [31], this strategy enhances students’ knowledge levels about life and positively contributes to their understanding of social phenomena depending on the type of text used in the practice of this strategy. It can be said that the close reading strategy has a significant effect on the development of sub-skills related to life skills of teacher candidates, such as coping with emotions and stress, empathy and self-awareness, decision-making and problem-solving skills, creative thinking and critical thinking skills, and communication and interpersonal relationships. From these findings, it can be suggested that the close reading strategy is a reading strategy that develops preservice teachers’ life skills in all aspects. Qualitative findings from the research also support these conclusions. Interviews with preservice Turkish teachers suggest that this strategy contributes significantly to respect for differences, openness to new ideas and experiences, critical thinking, self-awareness, self-regulation, empathetic thinking, decision making and evaluation, communication skills and interpersonal harmony, and expressing thoughts. Deep thinking [19,86] and improved attention skills [29] can be used to obtain different perspectives, encouraging decision making and inference [35]. Similarly, Lapp et al. [31] found that this strategy contributes to students’ ability to conduct critical examinations. When these results are examined, it can be concluded that these skills specifically include 21st-century life and professional skills. In addition to the results of this strategy’s contributions to vital skills, particularly in terms of group work, communication, and revealing the effects of group reading, decision making, and problem solving, it can also be said to contribute significantly to the development of essential skills in individual and professional life. Gucluer’s [77] research concludes that the use of this strategy allows students to share their emotions and thoughts. LaRusso et al. [10] assert that close reading is positively related to students’ academic language understanding, developing social perspectives, and reasoning about complex problems, thus supporting the findings of this study. In this respect, pedagogical strategy education in reading serves the purpose of achieving positive changes in life. This education is not only about reading comprehension, academic success, or other variables, but also about achieving the primary goal of reading in life skills. It can be evaluated as an important result. It can be said that this strategy, which is recommended for use in classes [64,66] due to its contributions to the reading process [62,63,65], should be used more widely due to its effect on the development of life skills. Since deep reading is a necessary skill for success in every area of life, teachers should not assume that their students come to them with advanced reading skills, and each class should continue to teach and develop these skills at their level [12]. As these are lifelong skills that require learning and development for lifelong use, students need to reach a level where they can read challenging texts in an increasingly complex world and acquire these strategies and skills independently to interact with books [16].

According to another result obtained from the research, it can be said that the close reading strategy provides significant development in terms of individual innovativeness characteristics of prospective teachers. The development of individual innovativeness manifests itself as new ideas and achievements, presenting outputs such as ideas, practices, and projects [87]. In this application, it can be said that in-depth reading of the texts affected the development of individual innovativeness of prospective teachers and contributed to their coming up with new ideas. However, a study by Baki [88] suggests that weekly and monthly reading rates of preservice teachers do not affect individual innovativeness in terms of these two variables. Despite reading being a basic tool for acquiring knowledge, this discrepancy can be considered as an effect of performing this study with a new strategy like the close reading strategy. When evaluated in terms of dimensions of individual innovativeness, this strategy was found to ensure significant development in terms of resistance to change but did not create a significant impact in terms of the leadership in ideas, openness to experience, or risk-taking dimensions. This may be due to the nature of philosophical texts that focus on thinking about thinking. According to qualitative findings from the research, some teacher candidates indicated that this strategy contributed to leading new ideas within the context of individual innovativeness. When this is associated with the contribution obtained in terms of resistance to change in the quantitative part of this study, it can be evaluated that preservice teachers do not resist innovations and follow the new ideas proposed to them, rather than proposing new ideas themselves.

As the categories would suggest, the majority were categorized in the questioning category, which consists of individuals who tend to accept proposed new ideas by looking at similar results rather than producing their own ideas. However, it is suggested that innovative individuals are expected to be risk-takers [48]. Teachers, who play a key role in the education sector and influence change in society, are the keys to renewal and change [55]. Therefore, it can be said that the practice of the close reading strategy successfully guides the development of reading skills in teacher education. This situation highlights the importance of different strategies and experiences, demonstrating that it is important for preservice teachers to increase their knowledge of different strategies, methods, and technical knowledge in every learning field since studies demonstrate that teachers’ attitudes towards learning develop as individual innovativeness increases [89,90,91]. Another result obtained from this research is that the majority of preservice teachers were in the pioneer and questioning categories prior to the practice of the close reading strategy, but following the practice, they moved to the questioning and skeptical categories, with no teacher candidates remaining in the innovative or traditional categories post-practice. This situation can be attributed to the close reading strategy that prioritizes critical thinking and questioning. Baki [88] also found that the majority of preservice teachers who enjoyed reading books were in the questioning and skeptical categories. However, depending on the number of books read during the year, the number of questioners increased while skeptics decreased, leading them to approach innovations cautiously based on their reading. Many studies have also revealed that preservice teachers are classified in the questioning category [45,47,55,88,92,93,94,95,96,97]. These results highlight the necessity of innovative educational environments that will transform knowledge from the level of questioning to innovation in teacher education, as well as introducing innovative, different, and original methods, strategies, and techniques because education is a sector that influences change, is affected by change, and ensures the training of the social workforce by determining practices and policies for change [55]. Therefore, it is of great importance that teachers, who are the main actors of these institutions, are trained as innovative and pioneering individuals in innovation, which is only possible by familiarizing them with contemporary methods, strategies, and techniques.

The changes resulting from the close reading strategy in individual innovativeness categories grouped by gender revealed that female preservice teachers were in the questioning category both before and after the practice, whereas male preservice teachers transitioned from the pioneer category before practice to the questioning and skeptical categories after. Studies indicating that gender is not a determinant in individual innovativeness include those by Başaran and Keles [98], Korucu and Olpak [47], Ozgur [99], and Yenice and Yavasoglu [100]. Additionally, there are studies suggesting that males tend to be more innovative [101]. Baki’s [88] study, however, revealed that male preservice teachers were not innovative, and the rate of innovativeness among females was very low. While these differences aim to describe an existing situation, it can be said that the result is similar in terms of the effect of the close reading strategy application. This can be attributed to the nature of the close reading strategy, focusing on critical thinking skills by delving into the layers of the text and questioning its meaning. In other words, although it affects preservice teachers’ innovativeness, this effect manifests itself in the development of questioning. In this case, it can be evaluated that the doubts of the prospective teachers increase as they learn, and this increase can be interpreted as the limited meaning of the existing knowledge in reaching the truth. In another parlance, all learned information aims to reach one’s own truth and absolute truth. However, their current readings are not sufficient for this, indicating that they cannot clarify the information.

As a result of the interviews conducted with Turkish teacher candidates, it can be said that the close reading strategy contributes to reading skills in terms of the development of the skills of analyzing the text in depth, the importance of using different strategies in the reading process, questioning the source and accuracy of the information in the text, the development of reading comprehension skills, and the skills of asking questions about the text. Similarly, Gucluer [77] discovered that the close reading strategy contributed to primary school students’ reading processes by providing positive developments in sentence length, sentence structure, meaningful sentence formation, and vocabulary, and significantly improved their comprehension levels. Lo-Philip [102] found that digital close reading activities helped some students organize their reading process by independently breaking down and combining words they would normally skip or misread without attempting self-correction. Houck’s [28] research also revealed that the questions used in the close reading process contributed to students’ interaction with and understanding of a text. Research has shown that this strategy enhances comprehension [28,62,63,65,103] and increases levels of participation in reading [26] and deep understanding skills [103]. Lapp et al.’s [31] study found that it contributed to students’ interaction with a text, questioning the content and information provided, and critically examining a text. This strategy helps students read more slowly and tackle challenging passages [86], thus improving their attention skills and note-taking skills [29]. Roberts and Roberts’ [11] study in the context of sociology classes concluded that deep reading activities helped students with their own reading and understanding, leading them to realize ways to create their own meanings from the text. This result aligns with the findings of this study regarding questioning the source of information in the text and developing text analysis skills. One of the most important factors in the emergence of these results is the active interaction of students with the text and the formation of questions related to the text during the process. Moreover, Lo-Philip [102] discovered that it was a popular class activity, and Gucluer [77] found that students reported enjoying reading, experiencing increased motivation towards reading, and developing a positive attitude towards this strategy. This strategy is effective not only for reading but also for other language skills. It can be said that these results align with the view in this study that preservice Turkish teachers recognize the importance of using different strategies during the reading process. Based on these results, it can be stated that in the teaching of a complex and strategic skill like reading, students should be brought to a level where they can use metacognitive reading strategies, teaching the use of multiple strategies and selecting and using them according to their developmental and personal characteristics. Finally, Houck’s [28] research demonstrated that this strategy contributed to the development of comprehension and metacognitive skills. Therefore, to achieve deep reading, understanding, and learning, students should be guided to reach a level of attention and awareness that addresses various ways of meaning-making, rather than reward- and punishment-based assignments and practices during the reading process [11]. This is because language skills are integrated and interact with each other, as highlighted by Dollins [104], who suggests that close reading strategies positively contribute to in-depth writing based on children’s books read with the help of this strategy.

## 5. Recommendations

1. The close reading strategy can be utilized for the development of life skills and individual innovation skills.
2. The close reading strategy should be included in the curriculum starting from primary education and continuing to higher education, and its use should be extended to different levels across this range.
3. The effectiveness of the close reading strategy on comprehension of narrative texts and poetry, as well as informative texts, can be examined.
4. This strategy was implemented with preservice Turkish teachers, and its effects on different educational levels can be examined in future research.
5. This study examined the strategy’s contributions to life skills, individual innovativeness, and the reading process. Future research could also examine its effects on other language skills such as writing, speaking, and listening.

## Figures and Tables

**Table 1:** Experimental design used in this study.

Group	Pre-Test	Method	Post-Test
G_d_	O_1.1_	X	O_2.1_
	1 week	7 weeks	1 week

Note: Test group: O_1.1_: pre-tests applied to the test group; O_2.1_: post-tests applied to the test group, X: awareness training and practice of the close reading strategy.

**Table 2:** Characteristics of the study group.

Features		Distribution	
		*f*	%
Gender	Female	26	83.87
	Male	5	16.12
Age	18–20	18	58.06
	21–23	10	32.25
	24–27	3	9.67
Class	3rd grade	26	83.87
	4th grade	5	16.12

**Table 3:** Texts used in the experimental process of the close reading strategy.

Weeks	Texts
Week 1	*Eyes*
Week 2	*Keep Searching*
Week 3	*Antigone*
Week 4	*Lord of the Flies*
Week 5	*Knowledge is Power*
Week 6	*On Three Changes*
Week 7	*A Letter on Tolerance*

**Table 4:** Dependent group *t*-test results for life skills pre-test and post-test scores.

Life Skills	Group	N	$\bar{X}$	S	sd	t	*p*
Coping with emotions and stress	Pre-test	31	20.25	4.96	30	2.99	0.00
	Post-test	31	22.25	4.31			
Empathy and self-awareness	Pre-test	31	24.80	3.48	30	−2.81	0.00
	Post-test	31	26.77	3.73			
Decision making and problem solving	Pre-test	31	24.96	3.77	30	−5.44	0.00
	Post-test	31	27.90	3.34			
Creative and critical thinking	Pre-test	31	18.48	2.64	30	−2.90	0.00
	Post-test	31	19.90	3.44			
Communication and interpersonal skills	Pre-test	31	14.51	2.58	30	−4.27	0.00
	Post-test	31	16.41	1.90			
Total of the scale	Pre-test	31	105.03	14.63	30	−5.02	0.00
	Post-test	31	114.83	14.33			

**Table 5:** Dependent group *t*-test results for individual innovativeness pre-test and post-test scores.

Individual Innovativeness	Group	N	$\bar{X}$	S	sd	t	*p*
Resistance to change	Pre-test	31	2151	9.02	30	−2.28	0.03
	Post-test	31	25.80	5.04			
Thought leadership	Pre-test	31	17.90	5.49	30	−0.92	0.36
	Post-test	31	18.93	3.01			
Openness to experience	Pre-test	31	18.29	5.54	30	−1.10	0.27
	Post-test	31	19.35	2.04			
Risk-taking	Pre-test	31	6.80	2.31	30	0.43	0.66
	Post-test	31	6.58	1.54			
Total of the scale	Pre-test	31	64.51	6.81	30	−4.27	0.00
	Post-test	31	70.67	6.58			

**Table 6:** Distribution of pre-test and post-test scores by gender and individual innovativeness categories.

Categories		Innovativeness Categories				
		Innovative	Pioneer	Inquisitive	Skeptical	Traditional
		*f*	*f*	*f*	*f*	*f*
Pre-test	Male	3	6	1	1	3
	Female	2	3	8	2	2
	Total	5	9	9	3	5
Post-test	Male	-	-	7	7	-
	Female	-	-	13	4	-
	Total	-	-	20	11	-

**Table 7:** The effect of the close reading strategy on the development of reading skills.

Main Theme	Sub-Theme	*f*	%
Reading skills	Analyzing the text in depth	31	100
	Importance of using different strategies	12	38.70
	Questioning the source and accuracy of the information in the text	10	32.25
	Reading comprehension	8	25.80
	Questioning skills	6	19.35

**Table 8:** Contributions of the close reading strategy to preservice Turkish teachers’ life skills and individual innovativeness.

Main Theme	Sub-Theme	*f*	*%*
Life skills	Respecting differences	31	100
	Finding practical and different solutions to problems	30	96.77
	Critical thinking	30	96.77
	Self-awareness	26	83.87
	Self-regulation	24	77.41
	Empathic thinking	21	67.74
	Decision making and evaluation	18	58.06
	Communication skills and interpersonal harmony	19	61.29
	Multidimensional and deep thinking	16	51.61
	Collaborative and group work	14	45.16
Individual innovativeness	Leading new ideas	30	96.77
	Being open to new ideas and experiences	13	41.93

## Data Availability

The original contributions presented in this study are included in the article; further inquiries can be directed to the corresponding author.
